# Src Kinase Regulation in Progressively Invasive Cancer

**DOI:** 10.1371/journal.pone.0048867

**Published:** 2012-11-07

**Authors:** Weichen Xu, Nancy Allbritton, David S. Lawrence

**Affiliations:** 1 Department of Chemistry, The University of North Carolina at Chapel Hill, Chapel Hill, North Carolina, United States of America; 2 Division of Chemical Biology and Medicinal Chemistry, Eshelman School of Pharmacy, The University of North Carolina at Chapel Hill, Chapel Hill, North Carolina, United States of America; 3 Department of Biomedical Engineering, The University of North Carolina at Chapel Hill, Chapel Hill, North Carolina, United States of America; 4 Department of Pharmacology, School of Medicine, The University of North Carolina at Chapel Hill, Chapel Hill, North Carolina, United States of America; Innsbruck Medical University, Austria

## Abstract

Metastatic progression is a multistep process that involves tumor growth and survival, motility and invasion, and subsequent proliferation in an inappropriate environment. The Src protein tyrosine kinase has been implicated in many of the biochemical pathways that drive these behaviors. Although Src itself is only rarely mutated in human tumors, its aberrant activity has been noted in various cancers and suggested to serve as a barometer of metastatic potential. With these features in mind, we examined Src kinase regulation at the structural, enzymatic, and expression levels as a function of progressively invasive prostate cancer cell lines. Surprisingly, both total Src content and kinase activity decrease with increasing cell line aggressiveness, an observation that appears to be inconsistent with the well-documented role of Src in the signaling pathways that drive growth and invasion. However, we do observe a direct correlation between Src kinase *specific activity* (total Src kinase activity/total Src content) and metastatic aggressiveness, possibly suggesting that in highly aggressive cell lines, key signaling enzymes are globally recruited to drive the cancerous phenotype. In addition, although the expected enhanced phosphorylation of Src at Tyr-416 (activation site) is present in the most aggressive prostate cancer cell lines, unexpectedly high phosphorylation levels at the Tyr-527 inhibitory site are observed as well. The latter, rather than representative of inhibited enzyme, is more indicative of primed Src responsive to local phosphorylated binding partners.

## Introduction

The Src protein kinase is the founding member of the Src kinase subfamily of nonreceptor protein tyrosine kinases. The catalytic activity of these enzymes is regulated, in part, via phosphorylation [1]. Specifically, full activation of Src is dependent upon phosphorylation at Tyr-416. By contrast, Tyr-527 phosphorylation inhibits catalytic activity, by promoting an intramolecular interaction with the enzyme’s SH2 (and SH3) domains. Indeed, the level of phosphorylated Tyr-527, as revealed by Western blot analysis or immunostaining, is often taken as a measure of inactive enzyme. For example, Src kinase activity was reported to be up-regulated in hormone-refractory prostate cancer as assessed by phosphorylated Tyr-416 content [2]. However, this interpretation is fraught with danger. Although the low *in vitro* catalytic activity of pTyr-527 Src is indisputable, the corresponding activity of the same enzyme in a biological environment is less certain. Upon interaction of the SH2 or SH3 domains of the Src enzyme with other proteins, the inhibitory pTyr-527 residue is released, which generates fully active pTyr-527 Src [3]–[5]. Indeed, this process is recapitulated by short pTyr-containing peptides that, upon binding to Src’s SH2 domain, disrupt the intramolecular inhibitory pTyr-527 interaction and thereby activate kinase activity [6]–[8]. Consequently, pTyr-527 status is not necessarily indicative of an inhibited enzyme *per se*, but rather of an enzyme that is potentially primed and ready to be “switched on” by interaction with an appropriate accessory protein. In short, high pTyr-527 Src levels could represent inhibited enzyme, activated enzyme, or some variant in-between. This uncertainty highlights the need to sample Src kinase *directly* via its ability to phosphorylate a target substrate. In addition to phosphorylation, the Src kinase is regulated at the protein level via ubiquitination and thus degradation by the proteasome-mediated pathway [9]. Consequently, in much the same way that phosphorylation status is a questionable harbinger of kinase activity, mRNA levels are not necessarily indicative of Src kinase content or activity.

Src is strongly implicated in the pathways that control many of the characteristic cell behaviors responsible for tumor invasion and progression. For example, Src mediates adhesion and cytoskeleton dynamics and thereby controls cell migration [10], [11]. Invasive migration is augmented by a Src-dependent epithelial-mesenchymal transition [12] and activation of matrix-degrading proteases [13]–[16]. Src phosphorylation of caspase 8 is antiapoptotic [17] whereas Src-mediated STAT activation promotes cell growth and survival [9]. In short, Src is implicated in a plethora of pathways that are up-regulated in many of the most aggressive and invasive forms of cancer. However, since Src is only rarely mutated in human tumors, aberrant Src behavior is most often a consequence of its abnormal regulation. We have examined Src kinase regulation at the structural, catalytic, and expression levels across a platform of prostate cancer cell lines that vary in behavior from noninvasive to highly invasive and from androgen-dependent to androgen-independent. Surprisingly, we found that Src catalytic activity and protein levels are significantly reduced in the most aggressive cell lines. By contrast, these aggressive cell lines display high Src specific activity and enhanced levels of phosphorylation at Tyr-527 (the so-called inhibitory site) relative to their less aggressive counterparts. These results are discussed in the context of the mechanism of Src activity in highly proliferative environments.

## Results

### Design and Synthesis of the Src Kinase Sensor

Based, in part, on a Src substrate sequence identified from an oriented peptide library [18], we constructed the fluorophore-labelled sequence: 5-FAM-Orn(Ac)-Glu-Glu-Glu-Ile-Tyr-Gly-Glu-Phe-Orn(Ac)-amide (**1**), where Tyr is the site of phosphorylation. In addition, ornithine (Orn) residues were placed on each terminus of the peptide sequence, in the event that the peptide proved to display poor selectivity for Src versus other protein kinases. We’ve previously shown that side chain modification of active site-directed peptides with unnatural substituents can dramatically enhance the selectivity for the target protein kinase (*vide infra*) [19]–[25]. Both the non-phosphorylated and the phosphorylated forms [5-FAM-Orn(Ac)-Glu-Glu-Glu-Ile-pTyr-Gly-Glu-Phe-Orn(Ac)-amide (**2**)] of the peptide were prepared via solid-phase peptide synthesis. 5-FAM (5-carboxyfluorescein) was chosen as the fluorophore for substrate and product detection by LIF.

**Figure 1:**
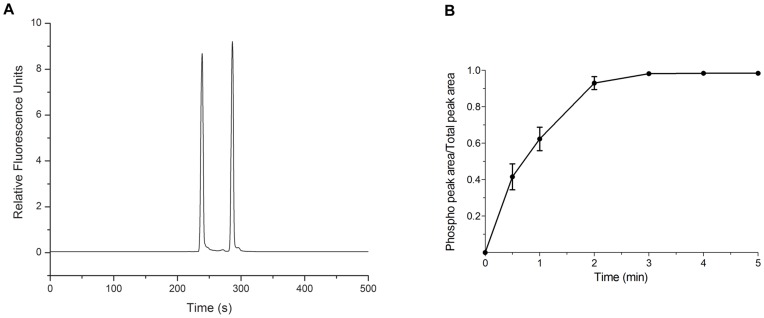
Assessment of Src kinase catalytic activity. (a) CE-LIF separation and visualization of the Src peptide substrate **1** and its chemically synthesized phosphorylated counterpart **2**. (b) Src kinase-catalyzed phosphorylation of peptide **1** as a function of time as assessed by CE-LIF.

The separation of the non-phosphorylated substrate and the phosphorylated product by CE-LIF is shown in Fig. 1A. A 1∶1 mixture of the peptides was loaded into the capillary. Under the separation conditions described in Materials and Methods, substrate peptide **1** emerges at 240 sec, whereas the phosphorylated product **2** appears at 290 sec. Growth of the latter was monitored, as a function of time, by incubating the substrate (**1**) with active Src kinase. The 290 sec peak was confirmed to be the phosphorylated product by spiking with synthetic phospho-peptide **2**. The kinetics of phosphorylation was obtained by evaluating collected time point samples via CE-LIF and quantifying the extent of phosphorylation via integration of the individual peaks. Peptide **1** phosphorylation is essentially complete within 3 min under the assay conditions that employed pure Src enzyme (Fig. 1B). By contrast, subsequent experiments in cell lysates (*vide infra*) proceed at a more leisurely pace. In the latter case, initial rate kinetics (<10% peptide phosphorylation) were acquired within 10 min of cell lysate incubation.

### Stability of the Src Kinase Peptide Substrate 1 in Prostate Cell Lysates

Peptides commonly suffer protease-catalyzed degradation in cells as well as in cell lysates. Consequently, prior to examining Src kinase-catalyzed phosphorylation of peptide **1** with lysates, we monitored the stability of the peptide as a function of time. Cells were lysed using Pierce’s M-PER mammalian lysis buffer and, in the absence of ATP and Mg^2+^ (i.e. no observable kinase activity), the lysate was added to peptide **1**. Only approximately 10% of the peptide is degraded after 1 h (Table S1). Since the phosphorylation rate is measured within 10 min of incubation (Fig. S1), the peptide is sufficiently stable for our needs.

### Assessment of Src Activity in Prostate Cell Line Lysates

**Figure 2:**
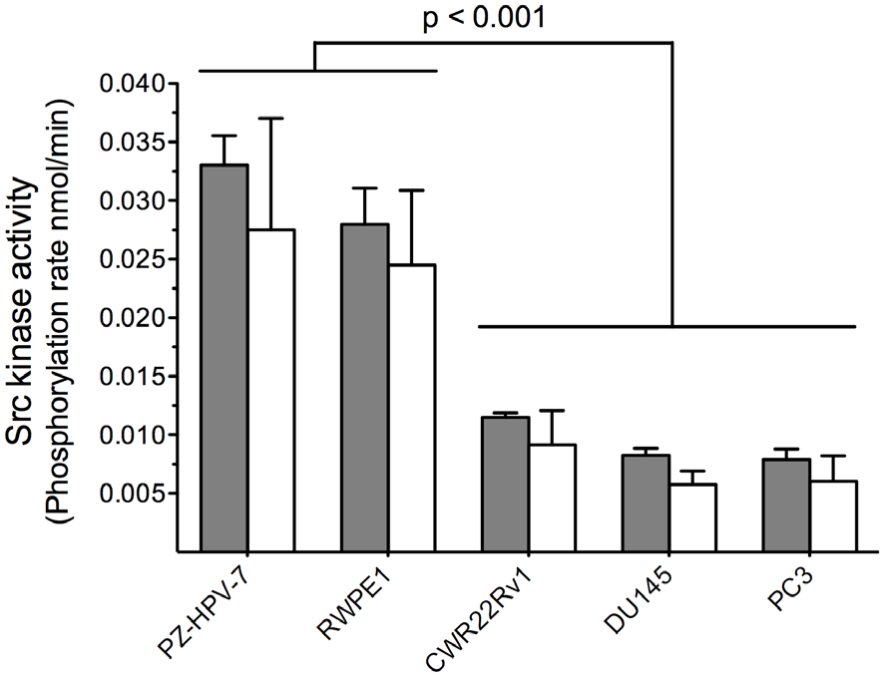
Src kinase activity in non-invasive (PZ-HPV-7, RWPE1), invasive (DU145, PC3), and androgen-independent (CWR22Rv1) cell lines. Grey bars are phosphorylation rates of peptide 1 by whole cell lysates (normalized by total protein extract amount). White bars are phosphorylation rates by cell lysates due to Src kinase alone after subtracting non-Src background phosphorylation of 1. Comparison between non-cancer (PZ-HPV-7, RPWE1) and aggressive cancer cell lines (CWR22Rv1, DU145, PC3) showed significant lowered levels of Src kinase activity associated with the later (p<0.001). All experiments were performed at least in triplicate. Error bars are SEM.

Our initial studies focused on the non-invasive immortalized normal prostate epithelial cell lines PZ-HPV-7 and RWPE1 and the highly invasive CaP cell lines DU145 and PC3. We also investigated Src activity in the non-metastatic CWR22Rv1 cell line, which exhibits androgen-independent growth, a characteristic behavior of advanced stage CaP [26] correlated with the Src kinase [2]. We assessed the initial rate kinetics (i.e. <10% of product formation) of peptide phosphorylation (Fig. S1) and were surprised to find that Src kinase activity (as a function of total protein extract amount) is significantly lower in aggressive prostate cancer cell lines (CWR22Rv1, DU145 and PC3) than the non-cancer prostate cell lines (PZ-HPV-7 and RWPE1) (Fig. 2; grey bars; P<0.001). This general trend is at variance with the general notion that high Src activity correlates with prostate cell line aggressiveness (i.e. invasive behavior and androgen-independent growth). Previous reports have noted that high levels of pTyr-416 are present in the most aggressive CaPs and it has been assumed, throughout the literature, that pTyr-416 and Src activity directly correlate with one another. One possible conclusion derived from our data is that the generally accepted notion that pTyr-416 levels serve as a barometer of Src activity is faulty. However, we investigated whether there might be alternative explanations for the unexpected relationship between Src activity and cell line aggressiveness. One possibility is that the Src substrate **1** is phosphorylated by other protein tyrosine kinases thereby rendering the apparent inverse relationship between Src activity and cell aggressiveness misleading.

### Assessment of the Selectivity of Peptide 1 for Src in Prostate Cell Lines

We examined the Src-selectivity of **1** using CaP cell lysates depleted of Src (Fig. S2). Although Src-free cell lysates are able to phosphorylate peptide **1**, they do so to a much lesser extent (<30%) than with whole cell lysates containing Src (Fig. 2, white bars). The contaminating tyrosine kinases that may be responsible for the low level of peptide **1** phosphorylation in the absence of Src could be one or more of the eight other members of the Src family kinases (SFK). Indeed, several SFK family members, such as Fyn, Brk, Lyn Lck and Yes are known to present in prostate cell lines [27]. Nonetheless, given the relatively modest level of peptide **1** phosphorylation by Src-free lysates, we decided that peptide **1** is, for our present needs, sufficiently Src selective.

We also assessed the ability of the Src peptide **1**/CE-LIF method to detect Src activity by using a combination of two known Src inhibitors. Imatinib (marketed as Gleevec), which is used to treat chronic myelogenous leukemia and other cancers, is an Abl-selective inhibitor with demonstrated weak anti-Src activity. Imatinib blocks kinase activity by serving as a competitive inhibitor of ATP. Like imatinib, saracatinib (AZD0530) is a competitive inhibitor of ATP, but displays a high selectivity and robust potency for Src. For example, the *IC*_50_ values of imatinib and saracatinib with isolated Src enzyme are 24.4 µM [28] and 2.7 nM [29], respectively. Using DU145 cell lysates, we found that the potent Src inhibitor saracatinib blocks peptide **1** phosphorylation with a submicromolar *IC*_50_ (0.35±0.08 µM) whereas the correspondingly poor Src inhibitor imatinib displays an *IC*_50_ that is in excess of 100 µM (Fig. S3). These results are consistent with notion, exemplified by the Src depletion experiments that the Src peptide **1**/CE-LIF method serves as an efficient and selective Src activity detection modality. Src kinase activity was also assessed in live single cells, both in the absence and presence of imatinib and saracatinib (Fig. S3).

### Src Expression Levels and Phosphorylation Status in Prostate Cell Lines

Src activity is higher in non-aggressive cell lines and lower in aggressive cell lines (Fig. 2), which is inconsistent with the general notion that Src plays a key role in carcinogenesis, metastasis, and the transition to the androgen-independent state. However, it occurred to us that total Src content may vary from one cell line to the next, which could the apparent (but misleading) inverse relationship between Src activity and CaP aggressiveness.

**Figure 3:**
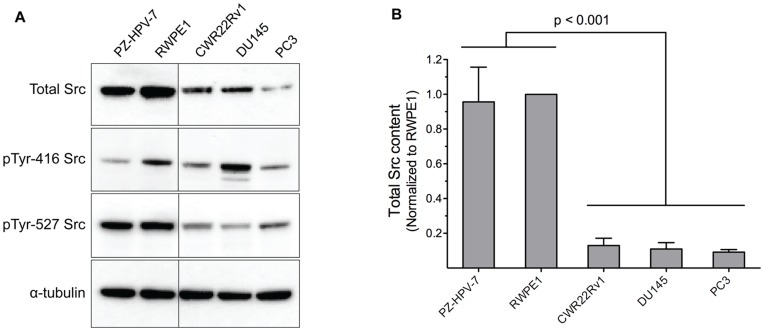
Src expression levels in non-invasive (PZ-HPV-7, RWPE1), androgen-independent (CWR22Rv1), and invasive (DU145, PC3) cell lines. (a) Prostate cell lysates were probed for total Src, pY416 Src and pY527 Src, where α-tubulin was used as the loading control. (b) Intensities of each band from the western blots were measured and normalized to the corresponding α-tubulin control, and then compared to cell line RWPE1 (RWPE1 as 1). Comparison between non-cancer (PZ-HPV-7, RPWE1) and aggressive cancer cell lines (CWR22Rv1, DU145, PC3) showed significant lowered levels of Src expression in the latter (p<0.001). Data are representative of at least three independent experiments and are mean with SEM.

**Figure 4:**
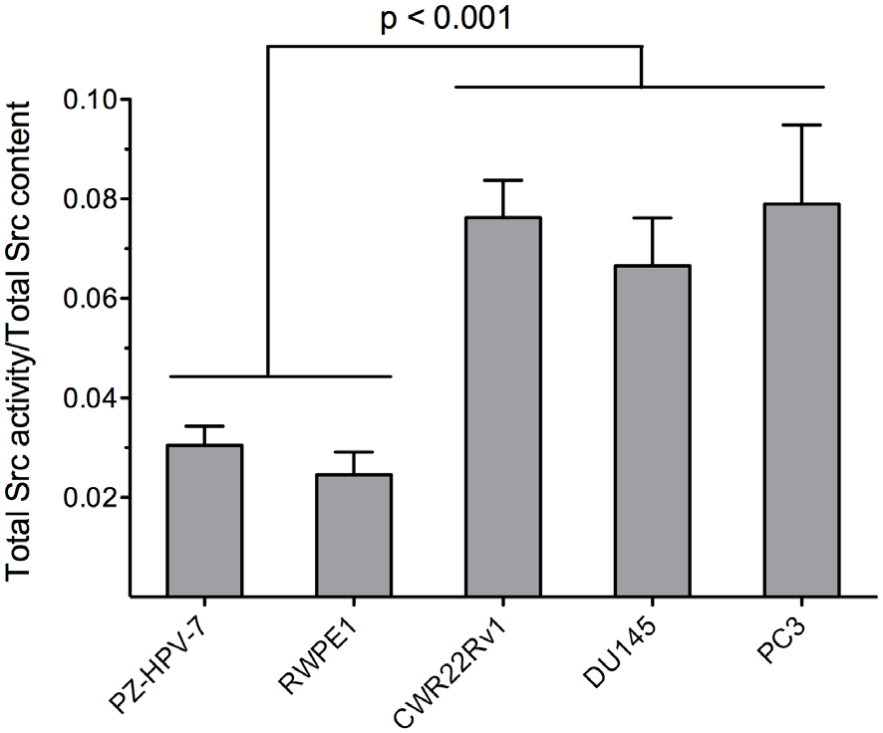
Src specific activity in non-invasive (PZ-HVP-7, RWPE1), androgen-independent (CWR22Rv1), and invasive (DU145, PC3) cell lines. Src kinase specific activity was calculated by dividing Src activity (Fig. 2) by total Src protein content (Fig. 3). Src specific activity is significantly higher in aggressive than in non-cancer cell lines (P<0.0001). Error bars are SEM.

Src content was quantified by western blot analysis (Fig. 3A) and normalized relative to α-tubulin. We found that the invasive cell lines, as well as the androgen-independent cell line, display less Src than the corresponding non-invasive, androgen-dependent cell lines (Fig. 3B, p<0.001). With this data in hand, we subsequently plotted Src specific activity (i.e. total Src activity/total Src content) versus cell line aggressiveness. The latter reveals Src specific activity is significantly higher in aggressive cell lines (CWR22Rv1, DU145, PC3) than in non-aggressive/normal cell lines (PZ-HPV-7, RWPE1) (Fig. 4, p = 0.0015).

**Figure 5:**
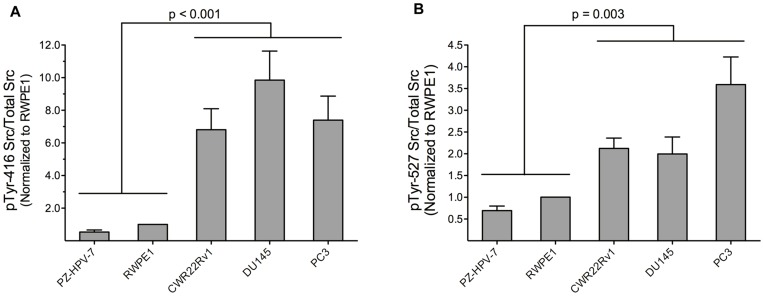
Src phosphorylation status in non-invasive (PZ-HVP-7, RWPE1), androgen-independent (CWR22Rv1), and invasive (DU145, PC3) cell lines. (a) pY416 levels were derived from the band intensities in the western blots (Fig. 3A), normalized to the corresponding α-tubulin control, and then compared to cell line RWPE1 (RWPE1 as 1). (b) pY527 levels were derived from the band intensities in the western blots (Fig. 3A), normalized to the corresponding α-tubulin control, and then compared to cell line RWPE1 (RWPE1 as 1). Data are representative of at least three independent experiments and are shown as mean with SEM. p values are indicated.

We also examined what relationship, if any, exists between the levels of pTyr-416 and pTyr-527 Src and Src specific activity. Phosphorylation at 416 is required for full activation of Src activity whereas phosphorylation at Tyr-527 is inhibitory due to the formation of an intramolecular interaction that blocks Src kinase activity [1]. pTyr-416 fractional levels (pTyr-416/total Src content) (Fig. 5A) is highly correlated with specific Src activity (Fig. 4) (r = 0.89), suggesting that pTyr-416 content is a good barometer of Src activity and cell line aggressiveness (Fig. 5A). However, as noted below, this correlation does not extend to all cell lines. By contrast, pTyr-527 content, a presumed indicator of inhibited Src, is more pronounced in aggressive cell lines (Fig. 5B). This observation is inconsistent with the general notion that activated Src correlates with cell line aggressiveness. In short, the phosphorylation status of Tyr-416 and Tyr-527, taken together, do not provide a consistent picture of Src activity across an array of CaP cell lines.

### Src Activity, Expression Levels, and Phosphorylation Status in RWPE1-Derived Prostate Cell Lines

**Figure 6:**
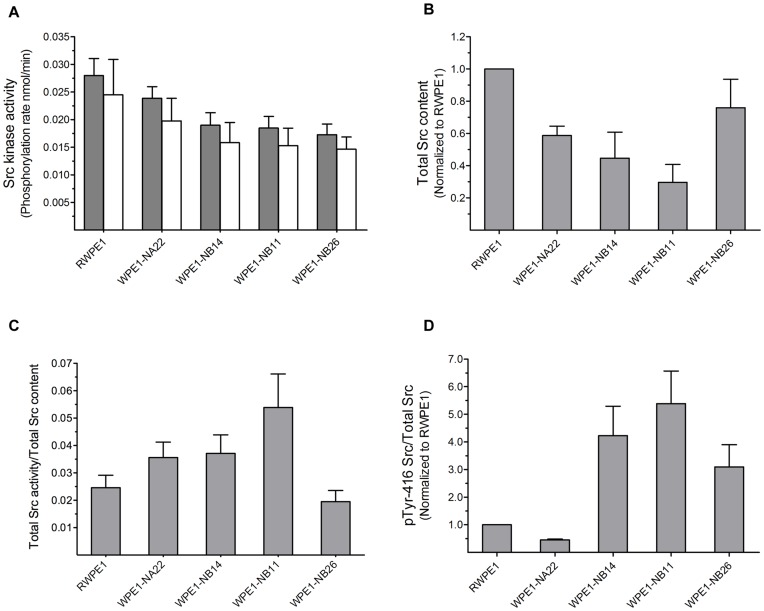
Src-catalyzed phosphorylation rates, Src protein content, and Src phosphorylation status in the RWPE1-derived cell lines. Increasing invasive ability is plotted along the x-axis. (a) Grey bars are phosphorylation rates of peptide 1 by whole cell lysates (normalized by total protein content). White bars are phosphorylation rates by cell lysates due to Src kinase alone after subtracting non-Src background phosphorylation of 1. (b) Total Src content as determined by western blot analysis (Fig. S5) (c) Src kinase specific activity as assessed by measured Src activity (Fig. 6A) divided total Src protein content (Fig. 6B). (d) pY416 levels were derived from the band intensities in the western blots (Fig. S4), normalized to the corresponding α-tubulin control, and then compared to cell line RWPE1 (RWPE1 as 1). Data are representative of at least three independent experiments and are shown as mean with SEM.

In addition to the standard prostate cell lines evaluated in Fig. 2, 3, 4, and 5, we evaluated a series of cell lines that were derived by exposure of RWPE1 to N-methyl-N-nitrosourea. Cell lines of increasing invasiveness have been identified via a series of *in vitro* and *in vivo* selection experiments: RWPE1, WPE1-NA22, WPE1-NB14, WPE1-NB11 and WPE1-NB26 [30]. This series displays Src kinase activity and aggressiveness correlations analogous to those described above for the standard prostate cell lines. First, total Src activity displays a significant decreasing trend (p = 0.018) as a function of increasing cell line aggressiveness (Fig. 6A). Second, with the exception of WPE1-NB26, total Src content also linearly decreases (p = 0.01) as a function of cell line aggressiveness (Fig. 6B and Fig. S5). Third, again with the exception of WPE1-NB26, specific Src kinase activity (Src kinase activity/total Src content) displays a significant linear increasing trend (p<0.001) as a function of increasing cell line aggressiveness (Fig. 6C). Finally, in sharp contrast to the results displayed in Fig. 5A, the correlation between pTyr-416 fractional levels (pTyr-416/total Src content) and Src specific activity is weak (r = 0.58), suggesting that it is dangerous to solely rely on pTyr-416 content as a barometer of Src activity (Fig. 6D). Interestingly, we do observe a very strong correlation with respect to fractional pTyr527 content and specific Src activity in RWPE1 series of cell lines (Fig. S6, r = 0.99), similar to that in Fig. 5B (r = 0.88).

## Discussion

Generic ELISA- [31] and γ-^32^P-ATP-based [32] methods are two of the most common strategies used for assessing activity with pure enzyme under *in vitro* conditions. In addition, fluorophore-labeled peptides [33]–[37], as well as GFP-based proteins [38], [39], have been described that exhibit a fluorescent response to phosphorylation, thereby allowing Src kinase activity to be continuously sampled in live cells via fluorescent microscopy. Although these technologies furnish a window into the biochemical basis of cell behavior, they are less readily translated into a routine, cross-platform methodology (pure enzyme, cell lysates, and intact cells) that can also be applied to the limited number of primary cells available in patient samples. In this regard, CE is an ultrasensitive method by which small amounts of analytes are separated and quantified, via the application of an electric field and subsequent detection of the analytes [40]. Since the Src kinase catalyzes the transfer of the γ-phosphoryl group from ATP to the tyrosine residue of the substrate, the charge difference between substrate and product should enable these species to be separated, visualized, and quantified via a shift in electrophoretic mobility [41]–[43]. Indeed, the fluorescein-substituted Src substrate **1** and its phosphorylated counterpart are readily differentiated upon exposure to both pure Src kinase (Fig. 1) and prostate cell lysates (Fig. 2). In the latter case, in nearly all cell lines, the vast majority of observed phosphotransferase activity (>70%) is due to the Src kinase (Fig. 2). The CE assay was further validated by examining the Src inhibitory potency of saracatinib and imatinib, where the latter is four orders of magnitude weaker (*IC*_50_ = 24.4 µM) than the former (*IC*_50_ = 2.7 nM) against pure enzyme [28], [29]. Consistent with these results, we observed a difference in inhibitory potency between saracatinib (*IC*_50_ = 0.35±0.08 µM) and imatinib (>100 µM) for Src in DU145 cell lysates analogous to the reported difference under simple buffer conditions. Finally, we investigated whether the CE strategy could be used to observe Src activity and inhibition in single cells (Fig. S4). DU145 cells were microinjected with Src substrate **1**, the cells subsequently incubated for 2 min, individually lysed using a focused Nd:YAG laser, and then the lysate from each cell separately electrophoresed. The formation of the phosphopeptide product was observed from cells that had not been preincubated with inhibitor (14±2% of total peptide content) or in cells preincubated with 1 µM of the weak inhibitor imatinib (*IC*_50_>100 µM; 15±3% of total peptide content). By contrast, no peptide phosphorylation was observed in cells that had been preincubated with 1 µM of the potent inhibitor saracatinib (*IC*_50_ = 0.35±0.08 µM).

With the ability to sample Src activity using purified enzyme, in cell lysates, and in single cells, we examined Src activity from noninvasive, invasive, and androgen-independent prostate cell lines (Fig. 2). Unexpectedly, we found that Src phosphotransferase activity is highest in the noninvasive cell lines, which appears to be contrary to the general notion that Src plays an important role in potentiating metastatic potential and the transition to and maintenance of androgen-independent growth in prostate cancer. However, it occurred to us that Src kinase levels might differ across cell lines. Once again, unexpectedly, we found that invasive and androgen-independent cell lines exhibit significantly less Src than their non-invasive androgen-dependent counterparts (Fig. 3). On the other hand, the ratio of Src activity to Src content (specific activity) revealed that aggressive and androgen-independent cell lines display a higher Src specific activity than non-aggressive androgen-dependent cell lines (Fig. 4). Furthermore, this trend holds for the series of N-methyl-N-nitrosourea cell lines derived from RWPE1 (from the peripheral zone of a normal human prostate). Specifically, we observed decreased total Src activity and Src content, and increased Src specific activity as a function of increasing cell line invasiveness (RWPE1<WPE1-NA22<WPE1-NB14<WPE1-B11) (Fig. 6A-B). We do note one exception to this correlation, namely the most aggressive RWPE1-derived cell line, WPE1-NB26. The latter has received significant attention due to its highly invasive phenotype [30], [44]–[53]. Using total Src content and specific activity as measures of aggressiveness, we would have assigned a non-invasive phenotype to WPE1-NB26 (i.e. similar to that of the parent cell line RWPE1). However, since this is clearly incorrect, the aggressiveness associated with this N-methyl-N-nitrosourea cell line may be due, at least in part, to mechanisms that are independent of Src signaling.

Why is the total Src content decreased in the most aggressive cell lines? One possible explanation is the known sensitivity of activated Src, relative to its non-activated counterpart, to ubiquitin-mediated degradation [54], [55]. Consequently, cell lines that are under intense activated Src pressure might, ironically, display lower Src content than less aggressive cells. In addition, two recent studies suggest a micro RNA (miR)-dependent mechanism by which Src levels are regulated. Bhatnagar *et al* have shown that the more invasive the prostate cell line the higher the miR-205 level [48]. miR-205 is known to downregulate Src expression [56]. Our observation that Src levels are lower in aggressive prostate cell lines is consistent with both ubiquitin and miRNA mechanisms. In addition, the higher specific activity in the most aggressive cell lines indicates that, in these cells, although there is less Src present, a greater fraction of it resides in the active form.

Src phosphorylation status has been assumed to correlate with activity where pTyr-416 is taken as active and pTyr-527 as inactive forms of the enzyme. With this in mind, we measured the levels of pTyr-416 via Western blot analysis and plotted pTyr-416 Src/total Src (fractional pTyr-416) versus cell line aggressiveness. As is evident from Fig. 5A and 6D, highly aggressive cell lines display a higher ratio of pTyr-416 to total Src than their nonaggressive counterparts. This is consistent with a recent report by Evans and colleagues demonstrating that the Src inhibitor saracatinib is most effective against prostate cell lines that have the highest ratio of active-to-total Src [57]. In the latter study, “active Src” was taken to be pTyr-416 Src. These investigators also reported that cells with the lowest (pTyr-416 Src/total Src) ratios express the most Src.

A high pTyr-527/total Src ratio should be, according to the conventional model, indicative of an overall lower fraction of active enzyme. However, we’ve found that this ratio is higher in aggressive lines as well (Fig. 5B and Fig. S5). One possible explanation for this unexpected result is the known ability of the pTyr-527 Src enzyme to be activated by phosphorylated peptides and proteins. In short, although pTyr-527 Src is inactive in its isolated, purified form, the opposite may be true when potential phosphorylated binding partners are present. Consequently, we conclude that pTyr-527 Src levels are not a useful barometer of inactive Src and, rather, may actually be a more appropriate prognosticator of Src activity. As an aside, we note that the Western blots employed in this study to detect Src phosphorylation status used large cell populations. However, recent advances, driven by CE technology, enable the phosphorylation status of proteins to be resolved and detected from as few as 25 cells [58]. Indeed, CE as a microscale Western blotting system has received significant attention [59]. Consequently, it may ultimately be feasible to simultaneously sample the levels of specific enzyme phospho-isoforms as well as enzyme activity in a few cells using CE technology.

There has been and continues to be a herculean effort to identify CaP markers that correlate with heritability as well as acquired somatic mutations. For example, more than 40 susceptibility loci have been assigned to approximately 25% of the heritable risk. [60] In addition, both coding region and whole genome analyses have been conducted to identify genetic aberrations that correlate with somatically acquired CaP. For example, a recent whole genome study was performed using tumor samples from patients with aggressive CaP. These investigators identified nearly 4000 somatic base mutations and 90 chromosomal rearrangements per tumor, as well as correlations between an array of rearrangement breakpoints and various epigenetic marks. [61] The authors note that a “spectrum of mechanisms direct prostate cancer genesis and progression” [61] and thus no single change in enzymatic activity is accountable for the onset, progression, and/or aggressiveness of the disease. For example, the complex genomic rearrangements prevalent in CaP are thought to impinge on the deletion and amplification of an array of genes encoding known tumor suppressors and oncogenes, respectively. Nonetheless, our data does suggest that Src specific activity may serve as a barometer of CaP aggressiveness. This is likely a consequence of the fact that Src itself is a key participant in pathways that mediate cell growth and motility, which in turn may reflect the massive and complex genomic structural changes prevalent in CaP. In addition, although we’ve found that pTyr-416 Src/total Src is a reliable indicator of Src specific activity, the results with pTyr-527 are, at first glance, unexpected. Phosphorylated Tyr-527 is generally considered to be reflective of inactive enzyme. However, we’ve found that the ratio of pTyr-527/total Src is a poor indicator of *in*active Src in CaP cell lines. Rather, the latter ratio appears to be a much better predictor of active Src. From a mechanistic point of view, this might reflect the presence of phosphorylated proteins that can associate with (via Src’s SH2 domain) and thereby rapidly activate the inhibited enzyme. Consequently, the activity status of pTyr-527 Src should be viewed as “primed for activity” rather than statically inactive.

In summary, we have developed a sensitive and selective Src kinase activity sensing system. Using a series of nonaggressive, aggressive, and androgen-independent prostate cell lines, we’ve found that total Src activity decreases as a function of increasing prostate cell line aggressiveness. However, this unexpected (and ultimately misleading) observation is a consequence of a corresponding decrease in total Src content in the more aggressive cell lines. When the latter is taken into account, a direct correlation exists between cellular aggressiveness and the fraction of cellular Src present in the active state.

## Materials and Methods

### Peptide Synthesis

Common analytical grade reagents were purchased from Fisher or Sigma-Aldrich. Common amino acids, O-(1H-6-chlorobenzotriazole-1-yl)-1,1,3,3-tetramethyluronium hexafluorophosphate (HCTU), N-Hydroxybenzotriazole (HOBT) and TGR resins were obtained from NovaBiochem. Fmoc-Orn(Aloc)-OH was obtained from Bachem. 5-carboxyfluorescein (5-FAM) was purchased from Anaspec. The non-phosphorylated peptide–resin Fmoc-Orn(Aloc)-Glu(OtBu)-Glu(OtBu)-Glu(OtBu)-Ile-Tyr(tBu)-Gly-Glu(OtBu)-Phe-Orn(Aloc)-amide-Resin and the phosphorylated peptide–resin Fmoc-Orn(Aloc)-Glu(OtBu)-Glu(OtBu)-Glu(OtBu)-Ile-Tyr(PO(OBzl)OH)-Gly-Glu(OtBu)-Phe-Orn(Aloc)-amide-Resin were synthesized using a standard Fmoc peptide synthesis protocol on the Prelude automatic peptide synthesizer (Protein Technologies). Amino acid (5 equiv), HCTU (5 equiv) and DIPEA (10 equiv) were mixed with resin in DMF and reacted for 10 min at room temperature. Fmoc deprotection was achieved using 20% piperidine in DMF for 20 min. The Aloc protecting group on the Orn residues was selectively removed with Pd(PPH_3_)_4_ (3 equiv) in CHCl_3_/AcOH/N-methylmorpholine (37∶2:1). The Orn residues were then acetylated using acetic anhydride/DIPEA (4∶1) and the N-terminal Fmoc protecting group was subsequently removed. The free N-terminus was then exposed to 5-FAM (5 equiv), HOBT (10 equiv) and N,N’-Diisopropylcarbodiimide (DIC, 15 equiv) in DMF overnight, and incubated with 30% piperidine for 15 min. The peptides were cleaved with TFA/H_2_O/TIS (triisopropylsilane) (95∶2.5∶2.5) and purified via HPLC (Waters), and their structures confirmed by ESI-Mass Spectrometry (Agilent Technologies). C_81_H_97_N_13_O_27_ (peptide **1**) Exact Mass calculated for 1683.7, found (ESI+) 843.0 (M+2H)^2+^, 562.5 (M+3H)^3+^ and C_81_H_98_N_13_O_30_P (peptide **2**) Exact Mass calculated 1763.6, found (ESI+) 883.0 (M+2H)^2+^.

### Western Blots

Primary antibodies Src (36D10): #2109, Yes: #2734, Phospho-Src Family (Tyr416): #2101, Phospho-Src (Tyr527): #2105, and α-tubulin (DM1A): #3873 were purchased from Cell Signaling Technology; anti-Fyn: 610163 and anti-Lck: 551047 were purchased from BD Pharmingen Biosciences; anti-Lyn (H-6): sc-7274 was obtained from Santa Cruz Biotechnology. Secondary antibodies were obtained from GE Healthcare. SDS-PAGE gel electrophoresis was performed using NuPage 4–12% Bis-Tris precast gel (Invitrogen), running at 200 V for 50–60 min. Gels were transferred using iBlot dry blotting system (Invitrogen) for 7 min. Blocking, antibody incubation, and washes were performed on SNAPid protein detection system (Millipore). Blots were detected by Amersham ECL Plus (GE Healthcare) and chemiluminescent images were obtained by the FluoChemFC2 system (Alpha Innotech). Band densitometry was performed using AlphaView software. The number of western blots reported for each data point in Figs. 3B, 4, 5, 6B–D was ≥3.

### Immunodepletion of Src Kinase from Cell Lysates

Src was captured by incubating cell lysate (500 µg)-antibody [5 µg; Src (36D10): 2109] mixtures with Pierce protein A/G agarose (Pierce Classic IP kit); and a BSA (5 µg) control was mixed with Pierce control agarose resin for 1 h at 4°C. Both treatments (with antibodies and with BSA) were separately repeated (3 h exposure time to antibodies/BSA and 1 h agarose-capture). Finally, the pre-cleared lysate was collected and concentrated using a Vivaspin 500 centrifugal concentrator, 10 kD (GE Healthcare) for western blot analysis and kinase assays.

### Capillary Electrophoresis

Capillary electrophoresis was performed on a ProteomeLab PA800 system equipped with a laser-induced fluorescence (LIF) detector (Beckman Coulter). The LIF was excited using the 488 nm argon laser. A fused-silica capillary (30/20 cm total/effective length, 50/360 µm inner/outer diameter) was used for separation (Polymicro Technologies). Prior to installation, capillaries were pretreated with 0.1 M NaOH for 12 h, distilled water for 1 h, 0.1 M HCl for 6 h and water again for 12 h. The samples were hydrodynamically injected and separated at 25°C at a constant voltage of 12 kV. The running buffer was 100 mM Borate/100 mM SDS buffer at pH 7.7. Before each run, the capillary was rinsed with 0.1 M NaOH, distilled water, and then the running buffer for 2 min under 20 psi pressure. Data was collected and analyzed using 32 Karat Software (Version 8.0, Beckman Coulter).

### Cell Culture

All cell lines were acquired from ATCC and grown in a humidified incubator at 37°C with 5% CO_2_. PZ-HPV-7, RWPE1, WPE1-NA22, WPE1-NB14, WPE1-NB11 and WPE1-NB26 cell lines were cultured in Keratinocyte Serum Free Medium (K-SFM) supplied with 0.05 mg/mL bovine pituitary extract (BPE) and 5 ng/mL epidermal growth factor (EGF) (all provided as a kit from Invitrogen), and 1% penicillin/streptomycin (P/S; Invitrogen). CWR22Rv1 cells were cultured in RPMI-1640 medium (Invitrogen) supplemented with 10% heat-inactivated fetal bovine serum (HI-FBS, Invitrogen) and 1% P/S. DU145 cells were cultured in Eagle’s Minimum Essential Medium (EMEM, ATCC) supplemented with 10% HI-FBS and 1% P/S. PC3 cells were cultured in F-12K medium (ATCC) supplemented with 10% HI-FBS and 1% P/S. Cell lysates were prepared as follows: confluent cells were first trypsinized and collected into 15 mL tubes in the presence of FBS-containing buffer or medium and then washed (3X) with ice cold DPBS buffer. The cells were lysed using M-PER mammalian protein extraction reagent mixed with 5/100 (v/v) Halt protease and phosphatase inhibitor cocktail EDTA-free (Thermo Fisher Scientific) for 10 min at 4°C. Cell debris was removed by centrifuging samples at 14,000 x g for 10 min at 4°C. The total protein concentrations were determined using Dc Protein assay (Bio-Rad).

### Protein Kinase Phosphoryl Transfer Activity via Capillary Electrophoresis

The pure enzyme phosphorylation assays were performed with a total volume of 40 µL from the following stock solutions: 30.9 µL of H_2_O, 0.5 µL of 1.14 mM substrate stock solution, 4 µL of 10X Src kinase reaction buffer (80 mM MOPS, 2 mM EDTA, 40 mM MgCl_2_, pH 7.0), 0.6 µL of 100 ng/µL active Src kinase (Millipore), 4 µL of 10 mM ATP. Final concentration of the assay solution was 8 mM MOPS, 0.2 mM EDTA, 4 mM MgCl_2_, 1.5 ng/µL Src kinase, 14.25 µM substrate, and 1 mM ATP. Assays were initiated by adding ATP. At each time point, 2 µL of the reaction mixture was removed and diluted with 8 µL 0.1 M HCl to stop the reaction. Cell lysate phosphorylation assays were performed similarly as described above using the following conditions: 2.5 µg/µL cell lysate, 6.75 mM Tris (pH 7.5), 1 mM ATP, 4 mM MgCl_2_, 57 µM substrate. Assays were initiated by adding substrate. Total protein content was used as the internal normalization of all cell extracts.

### Single Cell Capillary Electrophoresis

Custom made cell chambers were prepared by utilizing polydimethyl siloxane (PDMS, Sylgard 184) to glue a silicon O-ring to a glass coverslip (Fisher). The day prior to use, DU145 cells in suspension were diluted in 500 µL of complete media, plated on the chamber, and incubated in a 5% CO_2_ humidified incubator at 37°C. Single cell capillary electrophoresis was performed using a custom-made CE-LIF system, as described previously [62], [63]. Briefly, capillaries (30 µm inner diameter, 360 µm outer diameter, total length 38 cm with an effective length 21.5 cm) were conditioned with 0.1 M NaOH for 12 h, distilled water for 1 h, 0.1 M HCl for 6 h and distilled water again for 12 h. To initiate electrophoresis, the inlet reservoir was held at ground and a voltage of −10 kV was applied to the outlet. The electrophoretic buffer was 300 mM Borate buffer at pH 7.5. Each new chamber of cells was first washed with a constant flow of extracellular buffer (ECB; 135 mM NaCl, 5 mM KCl, 1 mM MgCl_2_, 1 mM CaCl_2_, and 10 mM HEPES, pH 7.4, 25°C) for 4 min. Microinjection of DU145 cells was performed using an Eppendorf Transjector 5246 and InjectMan system. 114 µM peptide **1** and 90X phosphatase inhibitor cocktail mixture (diluted from 100X phosphatase inhibitor cocktail 2, Sigma-Aldrich) was microinjected into a single cell (200 hPa, 1 s). For cells not treated with Src inhibitor, the ECB flow was kept on until 20 s after microinjection. For inhibitor-treated cells, the ECB flow was halted and replaced with 1 mL of inhibitor-containing ECB for 5 min. The ECB flow was turned on (40 s total) after microinjection to remove any residual peptides from the extracellular environment. The microinjected cell was incubated for 2 min prior to lysis by a focused Nd:YAG laser [64]. The lysate from the single cell was immediately loaded onto the capillary via electrokinetic injection and electrophoresed. Data was collected with custom software (LabVIEW 9.0.1, National Instruments) and analyzed by Origin (version 7.5, OriginLab Corporation).

### Statistical Analysis

Differences between non-cancer cells (PZ-HPV-7, RWPE1) and aggressive cancer cells (CWR22Rv1, DU145, PC3) were compared using the 2-tailed Student’s t-test, and a P-value <0.05 was considered statistically significant. Simple regression test with introduced indicator variable as 4,3,2,1,0 was performed to study the linear trend among RWPE1 series of cell lines, and a P-value <0.05 was considered statistically significant. Correlations between specific Src activity (total Src activity/total Src content) and phosphorylation status of Src (pTyr-416 Src/total Src or pTyr-527 Src/total Src) were compared using Pearson’s correlation. A correlation coefficient r>0.9 was considered very highly correlated; 0.7<r<0.9 was considered highly correlated; 0.5<r<0.7 was considered moderately correlated.

## Supporting Information

Figure S1 **Phosphorylation kinetics of peptide 1 by lysates from nine prostate cell lines.**
(TIF)

Figure S2 (a) Western blots of Src kinase from prostate cell line lysates and (b) validation of the antiSrc antibody.
(TIF)

Figure S3 **Normalized Src activity (where 1.0 is activity in the absence of inhibitor) in DU145 lysates versus log (a) [saracatinib] and (b) [imatinib] (µM).**
(TIF)

Figure S4 **Single cell analysis of Src kinase activity in DU145 cells by CE.** (a) Cells exposed to standard media, (c) standard media and saracatinib/AZD0530 (1 µM), and (e) standard media and imatinib (1 µM). All cells were subsequently microinjected with Src kinase sensor **1**, incubated for 2 min, lysed via sonication, the lysate loaded onto the capillary via electrokinetic injection, and electrophoresed. Electropherograms (a), (c), and (e) are representative of results where n = 5 for each experiment. The small peak at 160 s is a component present in the media. Peptide **1** is the large peak observed approximately 100 s prior to that of the corresponding phosphopeptide. Plot (b) is the control for (a): lysate from cells were exposed to standard media spiked with synthetically prepared phosphopeptide **2**; Plot (d) is the control for (c): lysate from cells exposed to standard media spiked with peptide **1** and phosphopeptide **2**; Plot (f) is the control for (e): lysate from cells exposed to standard media spiked with peptide **1** and phosphopeptide **2.** Controls were run either immediately prior to or following the corresponding experiments in (a), (c), and (e).
(TIF)

Figure S5 **Total Src content and phosphorylation status in prostate cell lines.**
(TIF)

Figure S6 **Src pY527 status in the RWPE1-derived cell lines with increasing invasive ability plotted along the x-axis.**
(TIF)

Table S1 Stability of the Src sensor in prostate cell lysates.
(PDF)
