# Supplementary figures and images for: Src Kinase Regulation in Progressively Invasive Cancer
Source: PLoS One. 2012 Nov 7;7(11):e48867. doi: 10.1371/journal.pone.0048867 (PMC3492248; doi:10.1371/journal.pone.0048867)

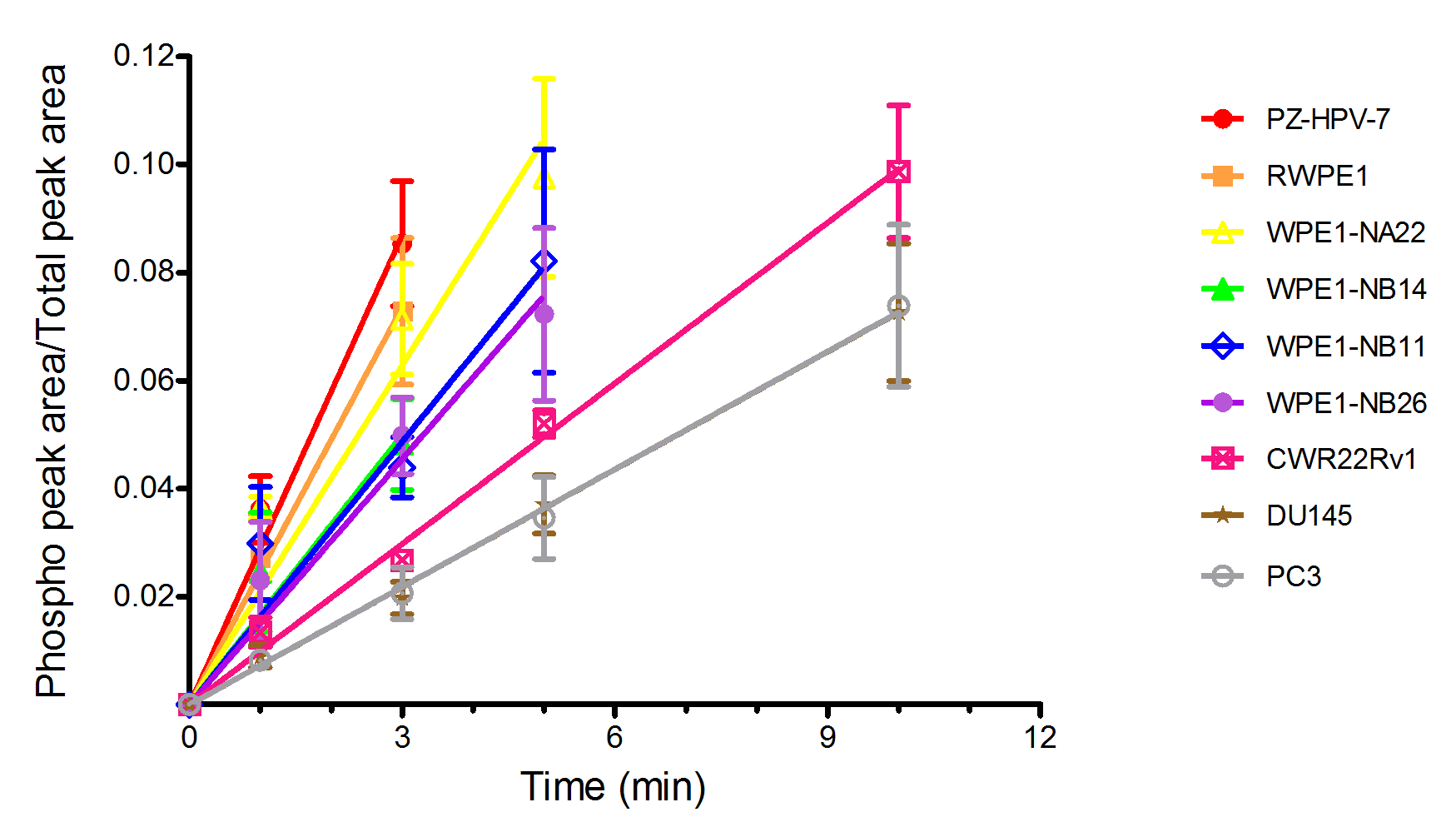

Supplement: Figure S1 — Phosphorylation kinetics of peptide 1 by lysates from nine prostate cell lines. (TIF) [file pone.0048867.s001.tif]

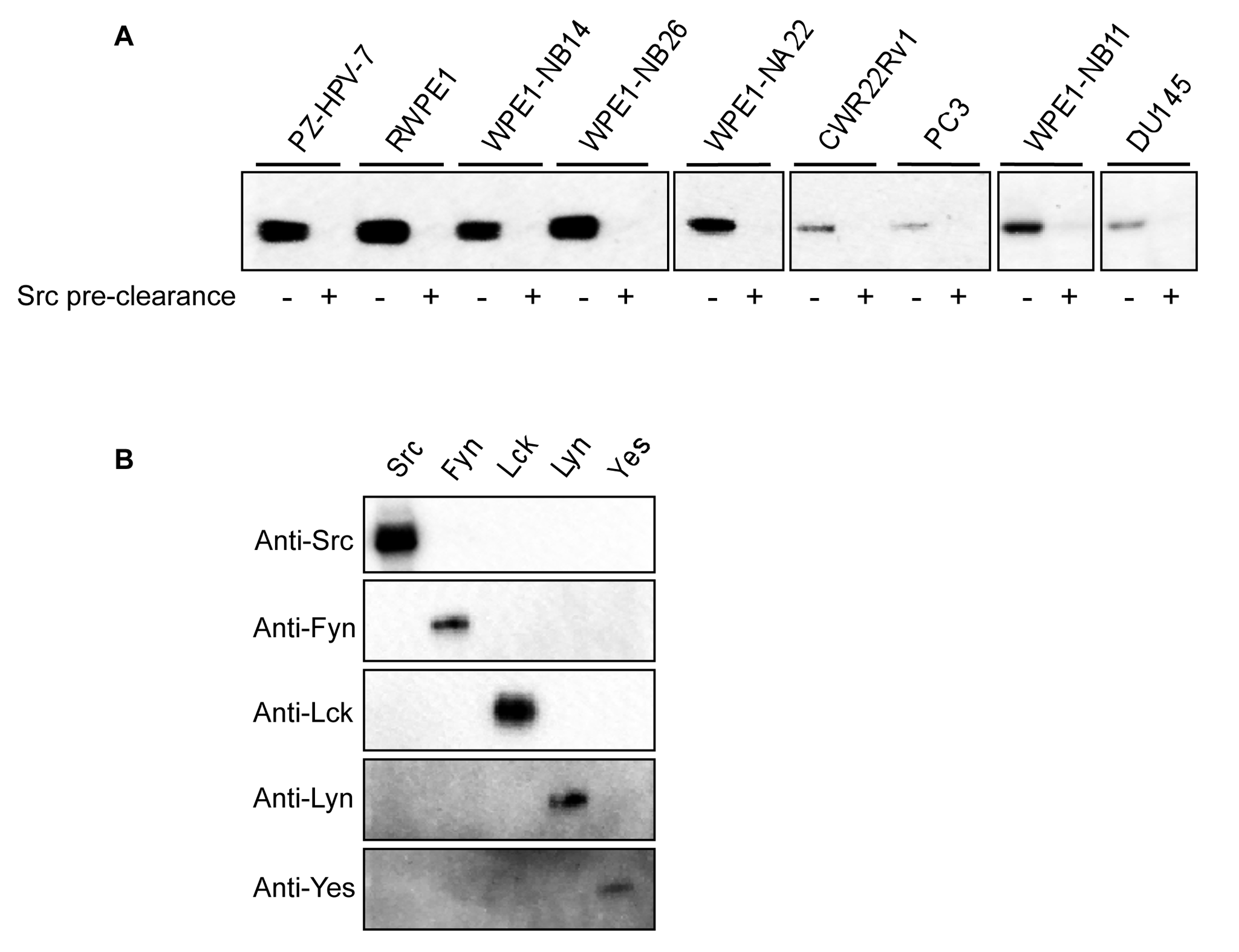

Supplement: Figure S2 — (a) Western blots of Src kinase from prostate cell line lysates and (b) validation of the antiSrc antibody. (TIF) [file pone.0048867.s002.tif]

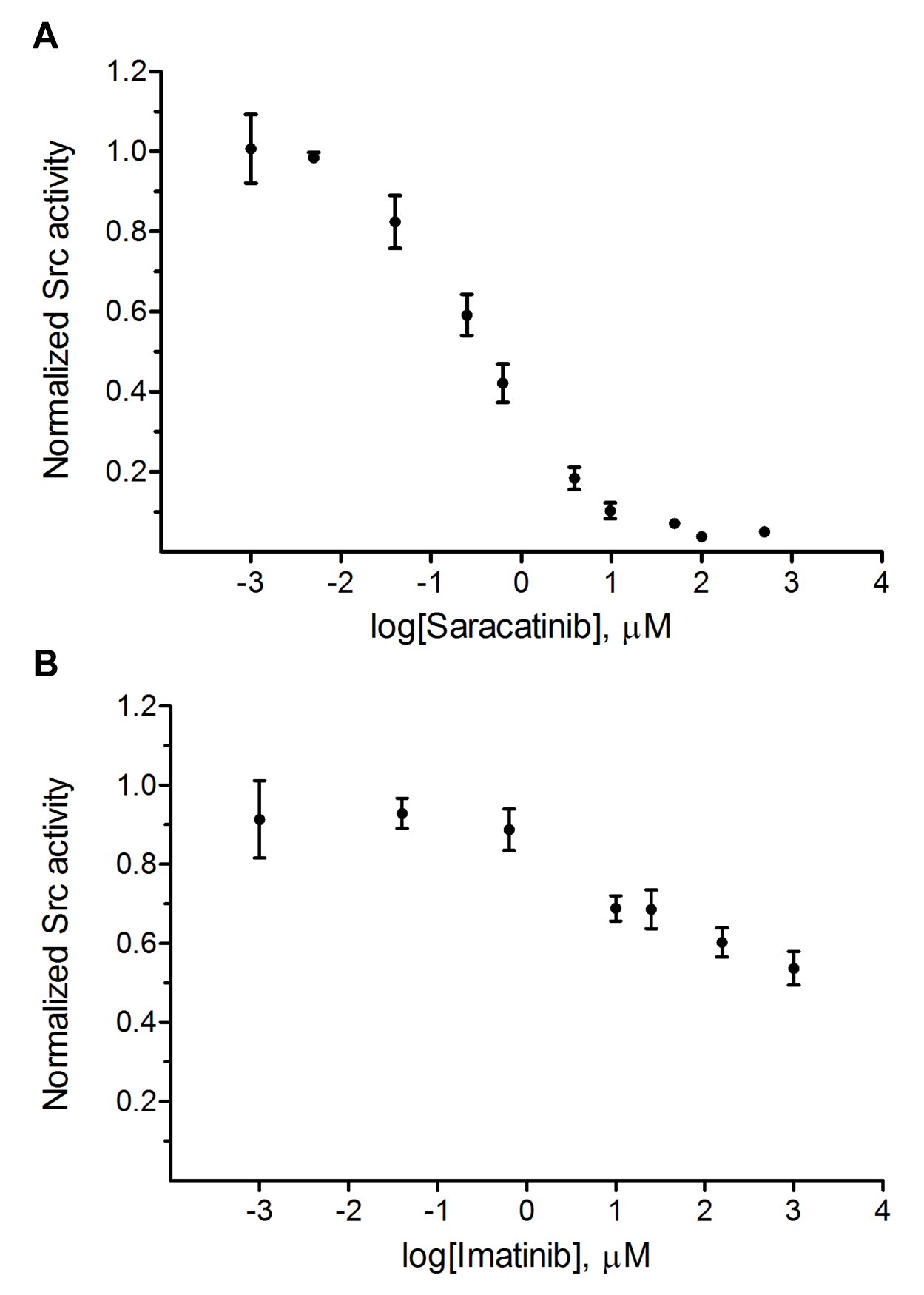

Supplement: Figure S3 — Normalized Src activity (where 1.0 is activity in the absence of inhibitor) in DU145 lysates versus log (a) [saracatinib] and (b) [imatinib] (µM). (TIF) [file pone.0048867.s003.tif]

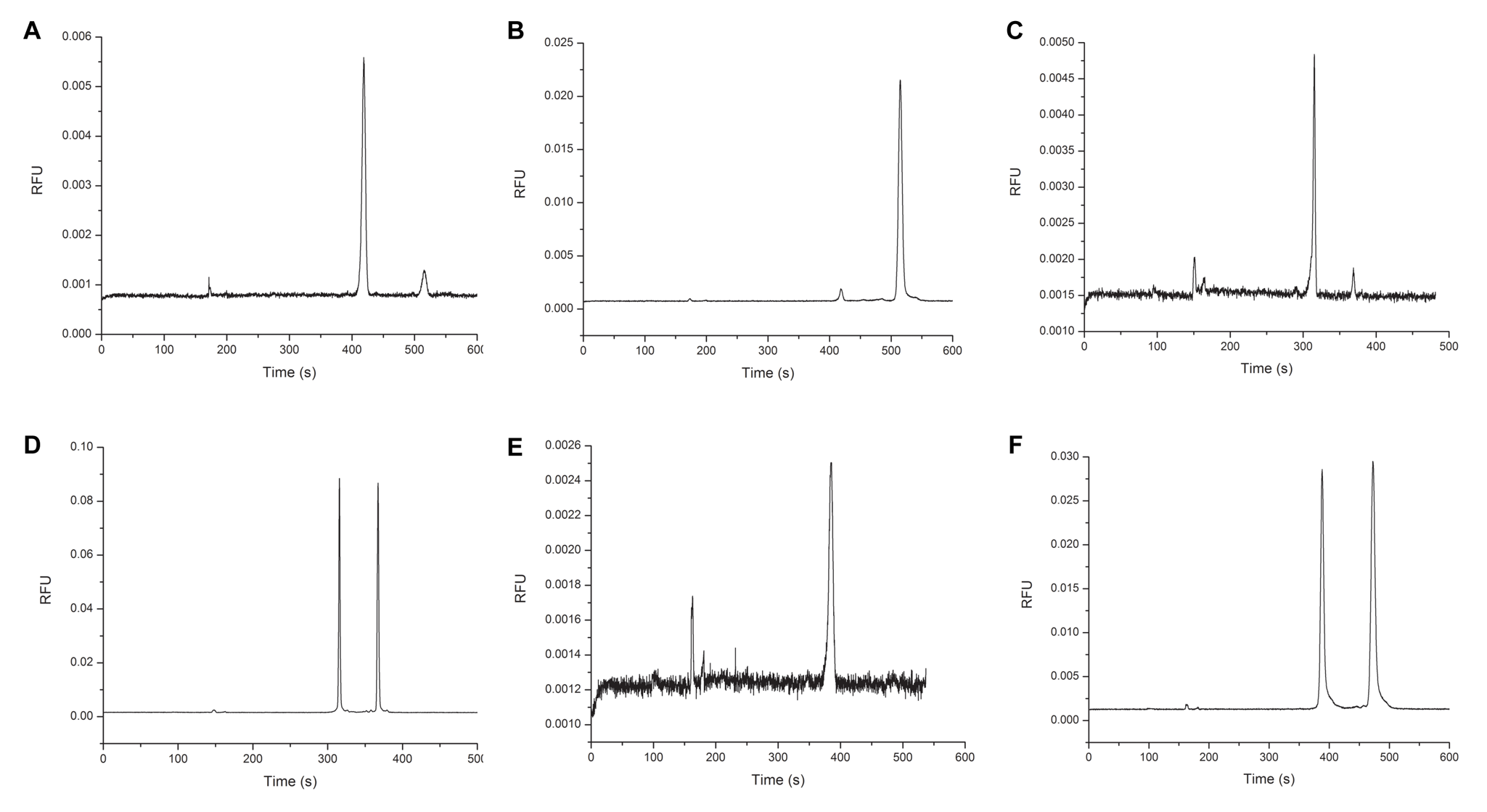

Supplement: Figure S4 — Single cell analysis of Src kinase activity in DU145 cells by CE. (a) Cells exposed to standard media, (c) standard media and saracatinib/AZD0530 (1 µM), and (e) standard media and imatinib (1 µM). All cells were subsequently microinjected with Src kinase sensor 1, incubated for 2 min, lysed via sonication, the lysate loaded onto the capillary via electrokinetic injection, and electrophoresed. Electropherograms (a), (c), and (e) are representative of results where n = 5 for each experiment. The small peak at 160 s is a component present in the media. Peptide 1 is the large peak observed approximately 100 s prior to that of the corresponding phosphopeptide. Plot (b) is the control for (a): lysate from cells were exposed to standard media spiked with synthetically prepared phosphopeptide 2; Plot (d) is the control for (c): lysate from cells exposed to standard media spiked with peptide 1 and phosphopeptide 2; Plot (f) is the control for (e): lysate from cells exposed to standard media spiked with peptide 1 and phosphopeptide 2. Controls were run either immediately prior to or following the corresponding experiments in (a), (c), and (e). (TIF) [file pone.0048867.s004.tif]

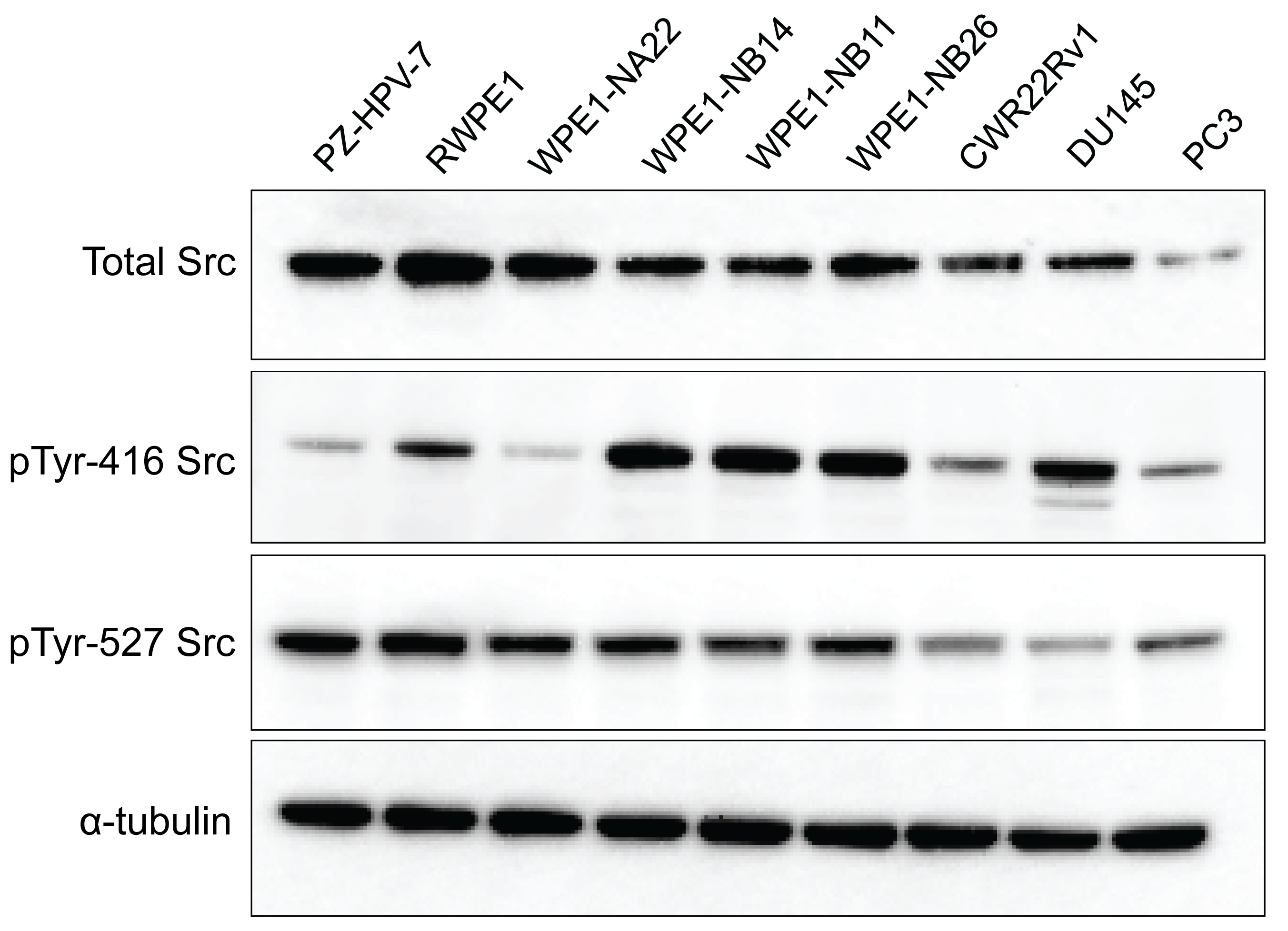

Supplement: Figure S5 — Total Src content and phosphorylation status in prostate cell lines. (TIF) [file pone.0048867.s005.tif]

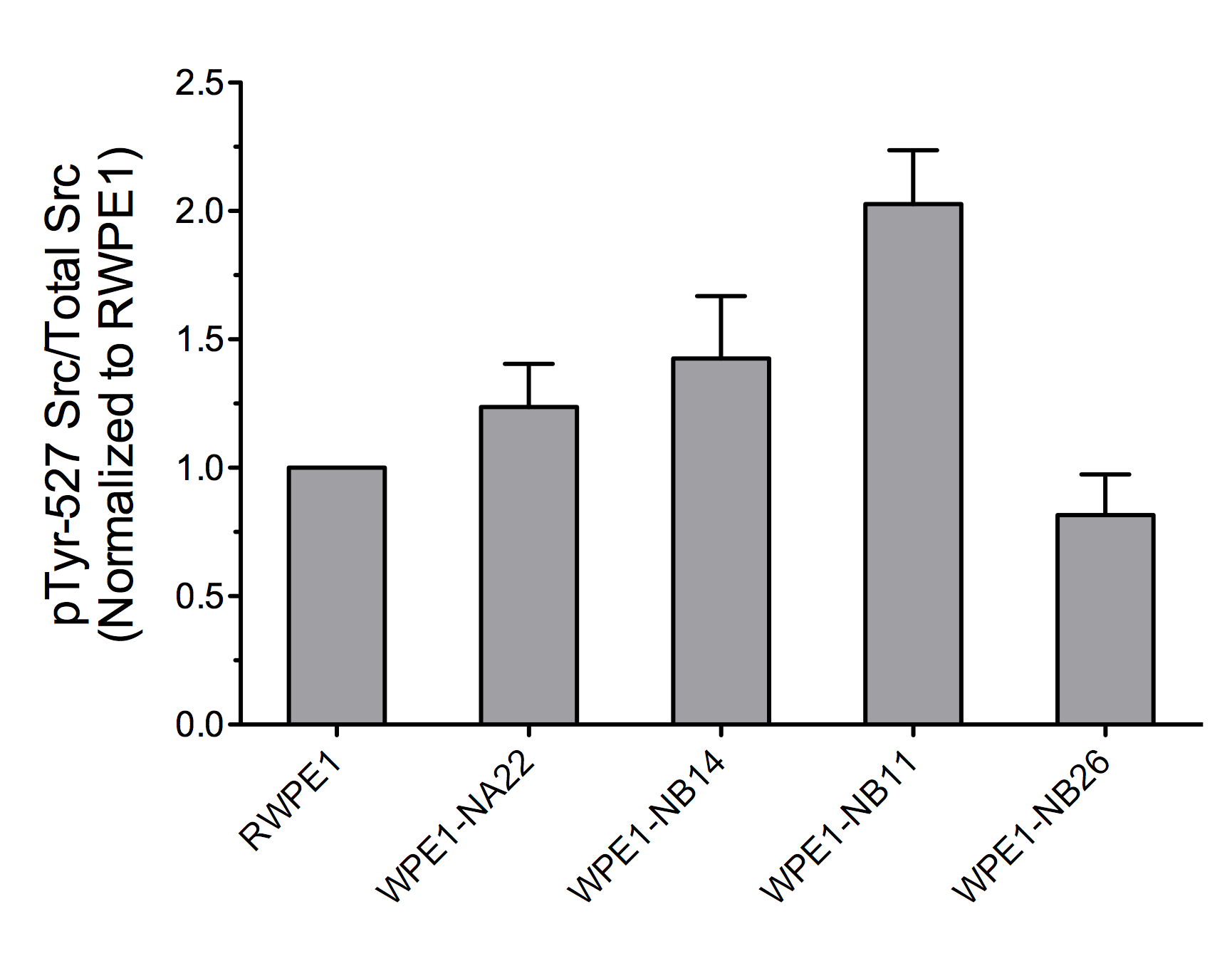

Supplement: Figure S6 — Src pY527 status in the RWPE1-derived cell lines with increasing invasive ability plotted along the x-axis. (TIF) [file pone.0048867.s006.tif]
