# Supplementary material for: Src Kinase Regulation in Progressively Invasive Cancer
Source: PLoS One. 2012 Nov 7;7(11):e48867. doi: 10.1371/journal.pone.0048867 (PMC3492248; doi:10.1371/journal.pone.0048867)
Supplement: Table S1 — Stability of the Src sensor in prostate cell lysates. (PDF) [file pone.0048867.s007.pdf]

**Cell Line**	**% Remaining Parent Peptide 1**
PZ-HPV-7	92.4 ± 0.2
RWPE1	91.0 ± 0.6
WPE1-NA22	92.4 ± 0.8
WPE1-NB14	91.4 ± 1.2
WPE1-NB11	89.8 ± 1.5
WPE1-NB26	91.0 ± 1.5
CRW22Rv1	91.2 ± 1.8
DU145	92.9 ± 0.5
PC3	89.2 ± 2.2
